# Regulatory Role of GSK-3**β** on NF-**κ**B, Nitric Oxide, and TNF-**α** in Group A Streptococcal Infection

**DOI:** 10.1155/2013/720689

**Published:** 2013-03-05

**Authors:** Yu-Tzu Chang, Chia-Ling Chen, Chiou-Feng Lin, Shiou-Ling Lu, Miao-Huei Cheng, Chih-Feng Kuo, Yee-Shin Lin

**Affiliations:** ^1^Department of Internal Medicine, National Cheng Kung University Medical College, Tainan 701, Taiwan; ^2^Institute of Clinical Medicine, National Cheng Kung University Medical College, Tainan 701, Taiwan; ^3^Department of Microbiology and Immunology, National Cheng Kung University Medical College, Tainan 701, Taiwan; ^4^Center of Infectious Disease and Signaling Research, National Cheng Kung University Medical College, Tainan 701, Taiwan; ^5^Institute of Basic Medical Sciences, National Cheng Kung University Medical College, Tainan 701, Taiwan; ^6^Department of Nursing, I-Shou University, Kaohsiung 840, Taiwan

## Abstract

Group A streptococcus (GAS) imposes a great burden on humans. Efforts to minimize the associated morbidity and mortality represent a critical issue. Glycogen synthase kinase-3**β** (GSK-3**β**) is known to regulate inflammatory response in infectious diseases. However, the regulation of GSK-3**β** in GAS infection is still unknown. The present study investigates the interaction between GSK-3**β**, NF-**κ**B, and possible related inflammatory mediators in vitro and in a mouse model. The results revealed that GAS could activate NF-**κ**B, followed by an increased expression of inducible nitric oxide synthase (iNOS) and NO production in a murine macrophage cell line. Activation of GSK-3**β** occurred after GAS infection, and inhibition of GSK-3**β** reduced iNOS expression and NO production. Furthermore, GSK-3**β** inhibitors reduced NF-**κ**B activation and subsequent TNF-**α** production, which indicates that GSK-3**β** acts upstream of NF-**κ**B in GAS-infected macrophages. Similar to the in vitro findings, administration of GSK-3**β** inhibitor in an air pouch GAS infection mouse model significantly reduced the level of serum TNF-**α** and improved the survival rate. The inhibition of GSK-3**β** to moderate the inflammatory effect might be an alternative therapeutic strategy against GAS infection.

## 1. Introduction

Group A streptococcus (GAS; *Streptococcus pyogenes*) is an important clinical pathogen in humans and induces a wide spectrum of clinical presentations including pharyngitis, erysipelas, necrotizing fasciitis, toxic shock syndrome, sepsis, and even mortality [1]. Numerous virulence factors of GAS, including surface molecules such as M protein, hyaluronic acid capsule, and C5a peptidase, or exotoxins, such as pyrogenic exotoxins, streptokinase, hyaluronidase, and streptolysins O and S, enable bacteria to resist phagocytosis in macrophages, escape from complement-mediated destruction, retard the influx of inflammatory cells, or trigger overreaction of immune system [2–6]. Despite advanced supportive care and effective antibiotic treatment, invasive GAS diseases are still responsible for at least 163,000 deaths each year in the world [2, 7, 8]. These facts highlight the urgent need for therapeutic strategies beyond effective antibiotic treatment. In patients with acute invasive GAS infection, the magnitude of elevation of IL-6 and TNF-*α* is closely related to the severity of systemic manifestations of the disease. Severe invasive cases suffering from toxic shock and/or necrotizing fasciitis have significantly higher frequencies of IL-2-, IL-6-, and TNF-*α*-producing cells in their circulation as compared to nonsevere invasive cases [9, 10]. Evidence suggests that the induction of cytokine production is, at least in part, due to superantigens of GAS. After the activation by superantigens, production of cytokines (e.g., IL-1, TNF-*α*, IL-6, and IFN-*γ*), which mediate shock and tissue injury, is initiated. Streptococcal pyrogenic exotoxin (SPE) B is able to cleave pre-IL-1*β* to release active form IL-1*β* [11]. Besides, peptidoglycan, lipoteichoic acid, and killed organisms are capable of inducing TNF production by mononuclear cells *in vitro* [12, 13]. Thus, clinical management to control the exacerbated inflammatory response caused by GAS infection may diminish collateral tissue damage and further reduce morbidity and mortality.

Glycogen synthase kinase-3 (GSK-3), a serine/threonine protein kinase, is involved in the regulation of many intracellular functions, including cell division, apoptosis, cell fate during embryonic development, signal pathways stimulated by insulin and many growth factors, and even the dysregulation of disease processes of cancer, diabetes, and neurodegenerative diseases [14–17]. In addition, GSK-3 is critical in either promoting [18] or repressing [19] the activity of NF-*κ*B which indicates its possible regulatory role in inflammation. Either GSK-3 inhibitors or siRNA could reduce the production of TNF-*α* and IL-6 and enhance IL-10 production in monocytes after stimulation by lipopolysaccharide (LPS) [20]. GSK-3*β* was also shown to regulate the STAT3-mediated IL-6 production in LPS-stimulated glial cells [21]. Furthermore, GSK-3 negatively regulated mycobacterium-induced IL-10 production and the subsequent IFN-*γ* secretion in monocytes [22]. In animal model of sepsis, treatment with GSK-3 inhibitors could suppress NF-*κ*B-dependent proinflammatory cytokine expression and provide protection from organ injury and endotoxin shock [20, 23, 24]. However, most of the previous studies investigating the role of GSK-3 in infection involved a LPS-induced sepsis model. Few studies have investigated the role of GSK in live bacterial infection, which is more representative of the clinical condition [25–28]. Also, less is known about the role of GSK in Gram-positive bacterial infection.

The macrophage is one of the frontline components in human innate immunity. It plays an important role in clearance of invasive pathogens through phagocytosis. Interference with macrophage function results in higher mortality rate in the GAS-infected animal model [29]. This implies an essential role of macrophages against GAS infection. In search of the possible role of GSK-3*β* in GAS-induced inflammatory response, we evaluated the activity of GSK-3*β* and subsequent inflammatory mediators in a mouse macrophage cell line and in the mouse model. Our results demonstrate that GAS infection induces GSK-3*β* activity, NF-*κ*B nuclear translocation, iNOS expression, and NO and TNF-*α* production. Inhibition of GSK-3*β* can negatively regulate the activity of NF-*κ*B and the production of NO and TNF-*α*. The effects of GSK-3*β* inhibitor were also observed in GAS-infected mice.

## 2. Material and Methods

### 2.1. Mice

BALB/c mice were purchased from the Jackson Laboratory, Bar Harbor, Maine, and maintained on standard laboratory food and water *ad libitum* in our animal center. Their progeny, ranging from 8to 10weeks of age, were used for experiments. The animal use protocol had been reviewed and approved by the Institutional Animal Care and Use Committee (IACUC). 

### 2.2. Bacterial Strain

*S.pyogenes* NZ131 (type M49, T14) was obtained from Dr. D. R. Martin, New Zealand Communicable Disease Center, Porirua. This strain does not contain phage-specific *spe*A and* spe*C genes.

### 2.3. Air Pouch Model of Infection

In our previous studies, we have established a mouse model of GAS infection using an air pouch [30, 31]. Mice were anesthetized by ether inhalation and then injected subcutaneously with 2 mL of air to form an air pouch on their back. Bacterial suspension was inoculated into the air pouch. Mice infected with GAS developed bacteremia and disseminated into the kidney, liver, and spleen. Mice died within few days (usually 3–7 days) after GAS infection. This model therefore serves as an animal model of sepsis. 

### 2.4. GAS Infection of Macrophages

Mouse macrophage cell line RAW 264.7 was cultured in DMEM with 10% FBS in 5% CO_2_ at 37°C. RAW 264.7 cells were seeded at 2 × 10^5^/well in 24-well plate containing medium without antibiotics. The next day, NZ131 cultures grown in TSBY were harvested at midlogarithmic phase and added to RAW 264.7 monolayers at different multiplicity of infection (MOI) of 10, 50, and 100. After 60 min of incubation at 37°C, nonadherent extracellular bacteria were eliminated by removing the culture medium and washing by PBS. Adherent extracellular bacteria were subsequently killed by incubation with fresh medium containing 10 *μ*g/mL penicillin G. At specific time points after infection, supernatants were collected for ELISA, and whole cell extracts were prepared for Western blot analysis.

### 2.5. ELISA

The levels of TNF-*α* in serum or cell culture supernatant were measured by ELISA kits (R&D system), according to the manufacturer's instructions. All measurements were carried out in triplicates. 

### 2.6. Western Blot Analysis

Whole cell extracts were separated using SDS-PAGE and transferred to polyvinylidene difluoride (PVDF) membrane. After blocking, blots were developed with rabbit antibodies against total and phosphorylated (Ser9) GSK-3*β*, total GS and phosphorylated (Ser641) GS, and mouse antibodies against iNOS. Mouse antibodies specific for GAPDH or *α*-tubulin were used for internal control. Finally, blots were hybridized with horseradish-peroxidase- (HRP-) conjugated goat anti-rabbit immunoglobulin G (IgG) or goat anti-mouse IgG, incubation with enhanced chemiluminescence (ECL) solution, and exposure to X-ray film.

### 2.7. Immunocytochemical Staining

Cells collected at various time points were fixed in 4% formaldehyde for 10 min. Adequately diluted anti-NF-*κ*B p65 antibodies were applied. After reaction with primary antibody at room temperature for 1 h, FITC-labeled secondary antibodies were applied for additional 1 h. DAPI was used for nuclear staining. The positive cells in three fields (under magnification ×200) of each culture were measured.

### 2.8. NO Analysis

After GAS infection for 24 h with or without GSK-3*β* inhibitors, supernatant of cell culture was collected. Then, 50 *μ*L Griess reagent was mixed with 50 *μ*L sample for measurement of nitrite level, according to the manufacturer's instructions.

### 2.9. Luciferase Reporter Assay

For the NF-*κ*B reporter assay, cells were transiently cotransfected with NF-*κ*B promoter-driven luciferase reporter (0.2 *μ*g) and 0.004 *μ*g of *Renilla* luciferase-expressing plasmid (pRL-TK; Promega) using the Gene Jammer transfection reagent (Stratagene). At 24 h after the transfection, cells were infected with NZ131 for 1 h and then replaced with medium containing antibiotics. Cells were then harvested for the luciferase assay (Dual-Glo; Promega). The firefly luciferase activity was normalized to the *Renilla *luciferase activity to evaluate transfection efficiencies.

### 2.10. Antibody and Reagent

Mouse monoclonal antibody specific for NF-*κ*B p65 was obtained from Chemicon International, Inc. Monoclonal anti-mouse iNOS was from BD Biosciences. Peroxidase-conjugated goat anti-mouse IgG and goat anti-rabbit IgG were from Invitrogen Corp. Antibodies against phospho-GSK-3*β* (Ser9), GSK-3*β*, phospho-glycogen synthase (GS) (Ser641), and GS were from Cell Signaling Technology, Inc. Antibody against GAPDH was from Millipore Corporation. Antibody against *α*-tubulin was from Santa Cruz Biotechnology Inc. Pyrrolidine dithiocarbamate (PDTC), 3-(2,4-dichlorophenyl)-4-(1-methyl-1H-indol-3-yl)-1H-pyrrole-2,5-dione (SB216763) and 3-[(3-chloro-4-hydroxyphenyl)amino]-4-(2-nitrophenyl)-1H-pyrrol-2,5-dione (SB415286) were from Tocris Bioscience. 6-Bromo-indirubin-3′-oxime (BIO), lithium chloride (LiCl) and ammonium chloride (NH4Cl) were from Sigma-Aldrich Co. Blocking antibodies specific for TLR2 and isotype-matched antibody control were from eBioscience, Inc.

### 2.11. Cell Viability

At 24 h after GAS infection and treatment with or without GSK-3*β* inhibitors, RAW 264.7 cells were flushed with culture medium in 6-well plates. Then, the whole culture medium was aspirated. The live and dead cells in culture medium were calculated directly under microscope after staining with trypan blue.

### 2.12. Mouse Survival Rate after GAS Infection

After inoculation with GAS into air pouch, various dosages of GSK-3*β* inhibitors were injected into the peritoneal cavity at different time points. The survival of mice after infection was observed every 24 h for 10 days.

### 2.13. Statistics

All statistics were performed using the two-tailed Student's *t*-test by the software of GraphPad Prism 5.01. The *P* values < 0.05 were considered significant. The mouse survival rate was analyzed by the Kaplan-Meier method.

## 3. Results

### 3.1. GAS Infection Induces the Activation of NF-*κ*B and the Increased Expression of iNOS and NO Production in RAW264.7 Cells

**Figure 1:**
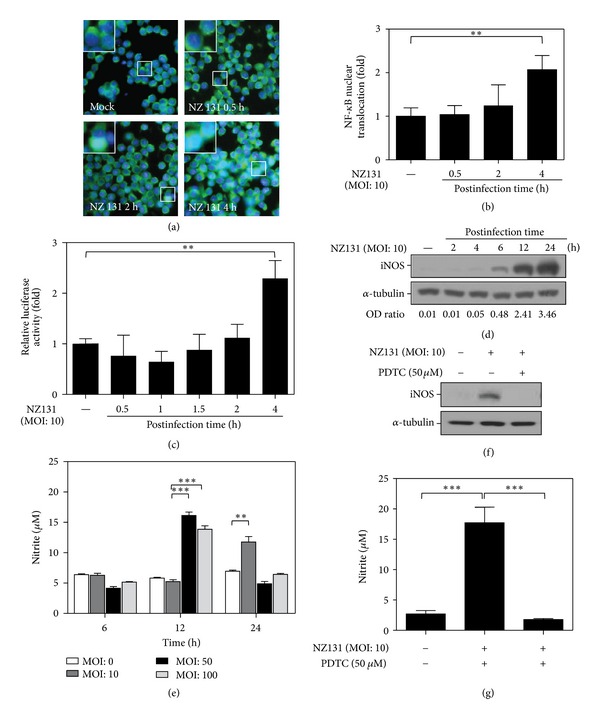
NZ131 infection induces the activation of NF-*κ*B and the increased expression of iNOS and NO in macrophages. (a) Using immunocytochemical staining for detection of NF-*κ*B p65 and DAPI for nuclear staining, the nuclear translocation of NF-*κ*B in RAW 264.7 cells (2 × 10^5^ cells/well in 24-well culture plate) after NZ131 infection (MOI: 10) at different time points was detected. Representative fields of NF-*κ*B translocation in RAW 264.7 cells are shown. (b) The bar chart graph is the summary of (a) for the ratio of the change of NF-*κ*B nuclear translocation after NZ131 infection at different time points. (c) RAW 264.7 cells were cotransfected with NF-*κ*B promoter-driven luciferase reporter and *Renilla* luciferase-expressing plasmid for 24 h. Then, RAW 264.7 cells were infected with NZ131 (MOI: 10) for 1 h. Luciferase activity was used to determine the dynamic change of NF-*κ*B activity 0.5–4 h after infection and the relative luciferase activity is the activity when compared with cells without NZ131 infection. (d) Western blot analysis was used to determine the expression of iNOS in RAW 264.7 cells stimulated by NZ131 (MOI: 10) for the indicated time points. (e) Griess reagent was used to determine the NO production in RAW 264.7 cells after treatment with different MOI of NZ131 (MOI: 0, 10, 50, or 100) at various time periods (6, 12, and 24 h after infection). (f) The expression of iNOS was determined by Western blot analysis with or without NF-*κ*B inhibitor, PDTC (50 *μ*M), for 6 h after NZ131 infection (MOI: 10). (g) The NO production was measured by Griess reagent 24 h after NZ131 infection (MOI: 10) with or without the concomitant treatment of PDTC. The data are means ± SD of the results obtained from three individual experiments. **P < 0.01; ***P < 0.001, comparisons between the indicated groups.

NF-*κ*B is a ubiquitous transcription factor and plays an important role in the host response to pathogenic organisms. The activation of NF-*κ*B can induce the expression of a number of molecules involved in inflammatory response, such as cytokines and iNOS [32, 33]. Infection of human respiratory epithelial cells with GAS induced the activation of NF-*κ*B [34]. To address if infection of macrophages with GAS could also induce the activation of NF-*κ*B, murine macrophage RAW 264.7 cells were infected with GAS NZ131 strain at MOI of 10 for various times. By using immunocytochemical staining and calculating the fold of NF-*κ*B nuclear translocation, the nuclear translocation of NF-*κ*B increased gradually after GAS infection and marked activation of NF-*κ*B was observed 4 h after infection (Figures 1(a) and 1(b)). Similar trend of NF-*κ*B activation was also observed when the activity was measured by luciferase reporter assay (Figure 1(c)).

To further evaluate the expression of iNOS and the subsequent production of NO, we determined the time kinetics and dose response of GAS by Western blotting and Griess reagent. The results revealed that GAS induced the expression of iNOS in a time-dependent manner (Figure 1(d)). The NO production was increased at 12 h with MOI of 50 or 100, and at 24 h with MOI of 10 (Figure 1(e)). To further clarify whether the iNOS expression and NO production were mainly through a NF-*κ*B-dependent pathway, RAW 264.7 cells were treated with GAS in the presence or absence of NF-*κ*B inhibitor, PDTC. Inhibition of NF-*κ*B activity caused suppression of iNOS and NO production (Figures 1(f) and 1(g)). These results reveal an essential role of NF-*κ*B in regulation of iNOS and NO expression in GAS infection.

### 3.2. GAS Infection Activates GSK-3*β* and Inhibiting GSK-3*β* Reduces the Expression of iNOS and the NO Production in RAW264.7 Cells

**Figure 2:**
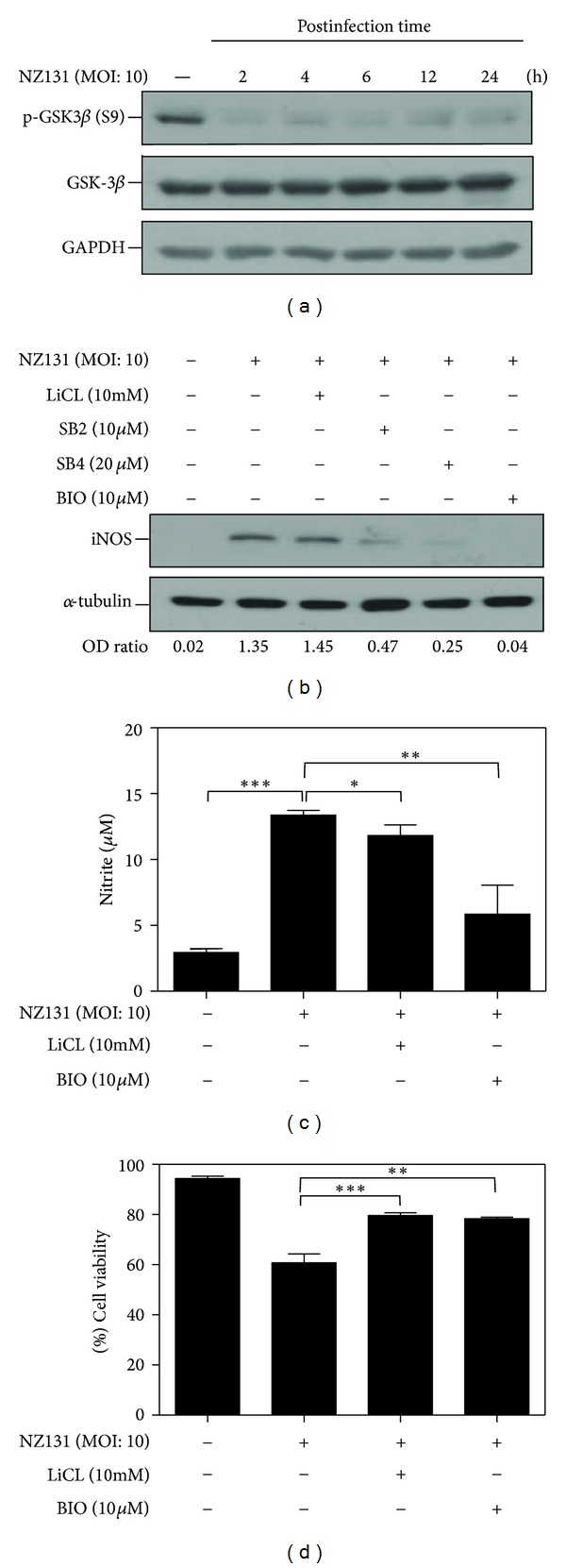
GSK-3*β* is activated in macrophages after stimulation by NZ131 and inhibiting GSK-3*β* reduces iNOS expression and NO production. (a) Western blot analysis was used to detect the expression of phospho-GSK-3*β* at Ser9 in RAW 264.7 cells (MOI: 10) at the indicated time points. (b) After pretreatment with various GSK-3*β* inhibitors (LiCl, SB216763, SB415286, and BIO), Western blot analysis was used to determine the expression of iNOS in RAW 264.7 cells at 6 h after NZ131 infection. (c) After NZ131 stimulation (24 h, MOI: 10), Griess reagent was used to determine the NO production in RAW 264.7 cells pretreated with GSK-3*β* inhibitors (LiCl and BIO). (d) Percentage of cell viability was determined 24 h after NZ131 infection with or without concomitant treatment with GSK-3*β* inhibitors (LiCl and BIO). The data are means ± SD of the results obtained from three individual experiments. *P < 0.05, **P < 0.01; ***P < 0.001, comparisons between the indicated groups.

Since GSK-3*β* was revealed to act upstream of NF-*κ*B [16, 18, 35, 36], we investigated whether GSK-3*β* may regulate NF-*κ*B during GAS infection and might be a potential therapeutic target. The dephosphorylation of GSK-3*β* at serine 9 was observed within 2 h after GAS treatment, which indicates the induced activation of GSK-3*β* in RAW 264.7 cells after GAS infection (Figure 2(a)). The phosphorylated GS, a GSK-specific substrate, also increased at 2 h after infection (Supplemental Figure 1 in supplementary material available online at http://dx.doi.org/10.1155/2013/720689). These results suggest that GAS infection could induce GSK-3*β* activation. When RAW 264.7 cells were stimulated with heat-inactivated GAS, there was only slight activation of GSK-3*β* (Supplemental Figure 2).

To further explore the role of GSK-3*β*, we found that the expression of iNOS was reduced by GSK-3*β* inhibitors (Figure 2(b)). Although the inhibition of iNOS expression was not apparent with lithium treatment as compared with SB compounds and BIO treatment, the NO production was significantly suppressed by lithium (Figure 2(c)). BIO could efficiently suppress the NO production. When measuring the cell viability under GAS infection with or without GSK-3*β* inhibition, GAS infection resulted in 29.23% cell death and treatment with GSK-3*β* inhibitors, lithium and BIO, could improve cell viability (Figure 2(a)). Thus, the reduction of NO production was not due to the reduced cell numbers. These results confirm that GSK-3*β* activation is involved in GAS infection and inhibition of GSK-3*β* reduces NO production, which implies the possible therapeutic application in GAS infection.

### 3.3. Inhibition of GSK-3*β* Activity Downregulates the NF-*κ*B Activation and TNF-*α* Production in GAS-Infected RAW 264.7 Cells

**Figure 3:**
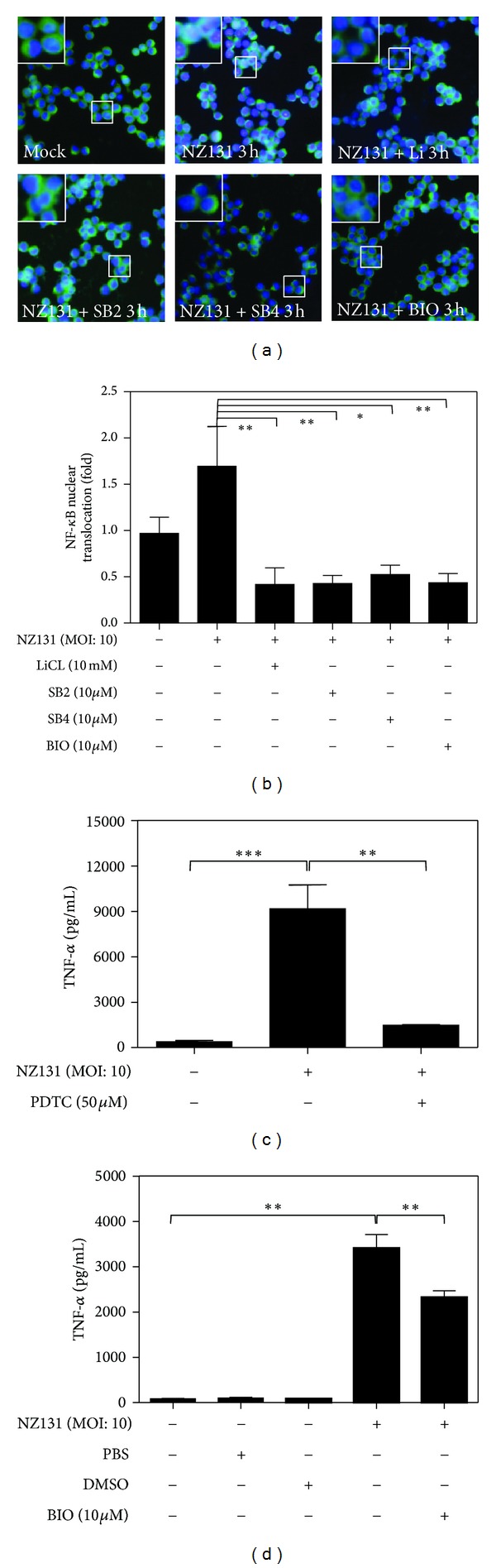
Inhibition of GSK-3*β* downregulates NF-*κ*B nuclear translocation and proinflammatory cytokine TNF-*α* production in macrophages after stimulation by NZ131. (a) Immunocytochemical staining for NF-*κ*B p65 and DAPI for nuclear staining were used to determine the nuclear translocation of NF-*κ*B in RAW 264.7 cells (2 × 10^5^ cells/well in 24-well culture plate) 3 h after NZ131 infection (MOI: 10). Representative fields of NF-*κ*B translocation in RAW 264.7 cells are shown. (b) The bar chart graph is the summary of (a) for the ratio of the change of NF-*κ*B nuclear translocation after NZ131 infection and pretreatment with various GSK-3*β* inhibitors (LiCl, SB2, SB4, and BIO). The data for each treated or untreated group is the average result calculated by six randomly selected fields in one experiment. ((c) and (d)) The concentrations of TNF-*α* in RAW 264.7 cell culture supernatant 24 h after NZ131 stimulation and pretreatment with NF-*κ*B inhibitor (PDTC) or GSK-3*β* inhibitor (BIO) were determined.

To further determine the essential role of GSK-3*β* in GAS infection, we examined the effect of GSK-3*β* inhibition on NF-*κ*B activation and TNF-*α* production. Using immunocytochemistry to detect NF-*κ*B p65 after GAS infection, nuclear translocation of NF-*κ*B was reduced after inhibiting GSK-3*β* by various inhibitors (Figure 3(a)). The ratio of NF-*κ*B nuclear translocation after GSK-3*β* inhibitor treatment was even lower than the basal level (Figure 3(b)). To investigate whether TNF-*α* production was also regulated by NF-*κ*B, we treated RAW 264.7 cells with PDTC under GAS infection. The TNF-*α* production was significantly suppressed by NF-*κ*B inhibition (Figure 3(c)). This implies that GAS induces TNF-*α* expression via NF-*κ*B activation. Furthermore, when treated with BIO and lithium, the production of TNF-*α* was also reduced in GAS infection (Figure 3(d) and Supplemental Figure 3). These results indicate that GSK-3*β* is involved in GAS-induced NF-*κ*B activation and TNF-*α* production, and inhibition of GSK-3*β* can lessen the GAS-induced inflammatory response.

### 3.4. GAS Internalization and TLR-2 Signaling Regulate TNF-*α* Production and iNOS Expression

GAS is known to survive and replicate in the macrophage and intracellular GAS can mediate NF-*κ*B activity [37]. Besides, cell wall components of Gram-positive bacteria can induce inflammatory response through TLR-2 [38]. We next determined whether GAS invasion or adherence to the plasma membrane can regulate TNF-*α* and iNOS expression. We found that either inhibition of endocytosis or blocking TLR-2 signaling can partially suppress TNF-*α* and iNOS expression (Supplemental Figures  3 and 4). These results suggest that GAS internalization and TLR-2 signaling may, at least in part, participate in the regulation of GAS-induced inflammatory response.

### 3.5. Inhibition of GSK-3*β* Activity Provides Protection of Sepsis Induced by GAS and Suppresses Serum TNF-*α* Level in Mouse Model

**Figure 4:**
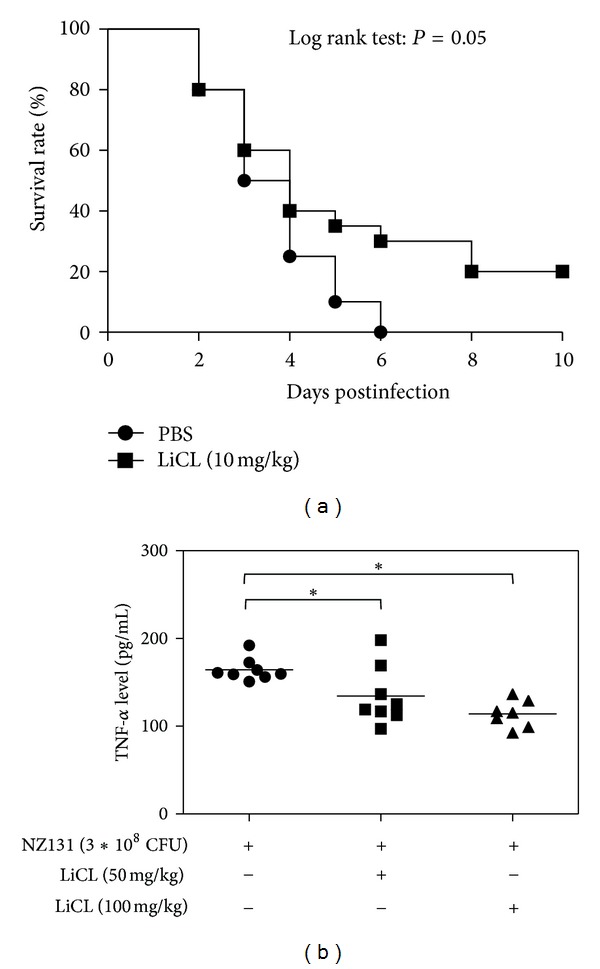
GSK-3 inhibitor, LiCl, can reduce the mortality induced by NZ131 infection in mice and suppress the TNF-*α* level in sera after NZ131 infection. (a) LiCl (10 mg/kg) was intraperitoneally administered immediately and at 12, 24, and 36 h after the inoculation of NZ131 (3 × 10^8^ CFU) into the air pouch created on the back of BALB/c mice (*n* = 20 per group). The Kaplan-Meier method was used to determine the difference of survival rate after NZ131 infection. *P* value was analyzed by log rank test (*P* = 0.05). (b) LiCl was given intraperitoneally once immediately after inoculation of NZ131 into air pouch and mice were sacrificed 24 h after infection when serum was collected for measurement of TNF-*α* (*n* = 8 in NZ131 alone group, *n* = 8 in LiCl 50 mg/kg group, and *n* = 7 in 100 mg/kg group).

Since the inhibition of GSK-3*β* led to reduced NF-*κ*B activation and TNF-*α* production *in vitro*, we next determined whether this protective effect via GSK-3*β* inhibition could be observed *in vivo*. Administration of lithium, a drug which has been used for bipolar disorder, improved the survival rate in the treated group (*P* = 0.05) (Figure 4(a)). Lithium treatment after GAS infection could significantly suppress TNF-*α* production (Figure 4(b)). Thus, inhibition of GSK-3*β* could reduce the level of TNF-*α* and increase survival in the GAS-induced sepsis model.

## 4. Discussion

**Figure 5:**
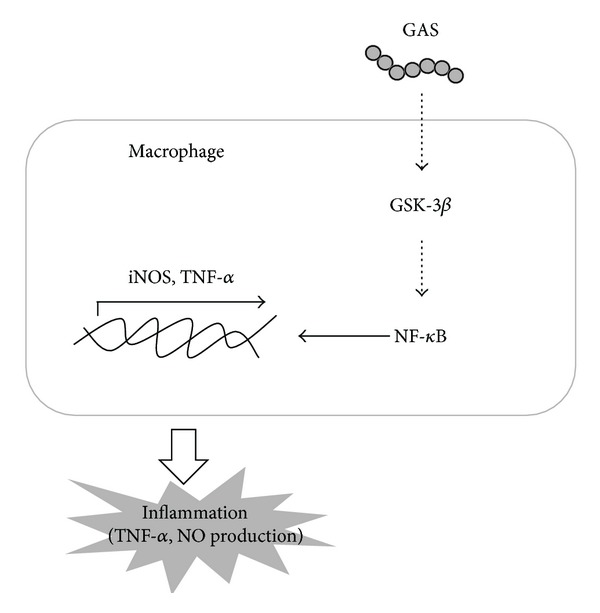
The signaling pathway of group A streptococcus-induced GSK-3*β* activation, which leads to increased NF-*κ*B nuclear translocation and upregulation of TNF-*α* and NO production in macrophages.

Group A streptococcus is an important human pathogen and carries a high clinical burden worldwide [7]. Reducing the related complications and mortality and morbidity caused by GAS infection remains a critical issue. GSK-3, a key enzyme in glycogen metabolism, is involved in many intracellular functions [15]. In recent studies of shock and inflammation [16, 20, 23, 36], GSK-3*β* inhibitors can not only suppress the production of proinflammatory cytokines but also increase anti-inflammatory cytokines. GSK-3*β* inhibitors also provide a survival advantage and can attenuate organ injury in animal models of sepsis. However, the protective effect of GSK-3*β* inhibition was mostly demonstrated in LPS-induced sepsis models and only a few studies explored the possible effect of GSK-3*β* inhibition against live bacteria, which is more similar to clinical conditions [26]. In the present work, we proved that GAS infection induced the activation of GSK-3*β* and increased nuclear translocation of NF-*κ*B, as well as iNOS expression and NO and TNF-*α* production. Inhibiting the activity of GSK-3*β* was able to downregulate the activation of NF-*κ*B and suppress the subsequent inflammatory mediators, including iNOS, NO, and TNF-*α* induced by GAS infection. This result is also consistent with previous findings that GSK-3*β* acts upstream of NF-*κ*B [16, 18, 35, 36] and the present study indicates that GAS activates macrophages through a GSK-3*β*-NF-*κ*B-dependent pathway (Figure 5). Previous report showed that heat-inactivated *Staphylococcus aureus* could also mediate GSK-3*β* activity [39]. In the present study, the GSK-3*β* activity was only slightly increased after stimulation with heat-inactivated GAS (Supplemental Figure 2). Whether GAS-induced GSK-3*β* activity could be related to some secreted proteins, in addition to cell wall components, remains to be explored. Furthermore, the identities of the upstream molecules or even the recognition receptor are not clear. Previous studies [40, 41] indicated that the recognition of GAS and subsequent signal transduction was MyD88 dependent. Thus, further studies are needed to clarify this crosstalk between GSK-3*β* and MyD88 and NF-*κ*B. 

GSK-3*β* is known to regulate the main eukaryotic transcription factor NF-*κ*B, which is involved in many intracellular processes. After activation of NF-*κ*B, its subunit of p50/p65 translocates from cytoplasm to the nucleus and initiates target gene transcription, including proinflammatory cytokines, chemokines, adhesion molecules, matrix metalloproteases (MMPs), and iNOS [42–44]. Various reports [16, 20, 45, 46] have shown that GSK-3*β* is able to affect NF-*κ*B activity by several different mechanisms: (1) through phosphorylation of I*κ*B, (2) facilitating translocation of p50/p65 to the nucleus and the binding to DNA, (3) phosphorylation of p65, or (4) through CREB. In our present work, we proved that NF-*κ*B plays an important role in GAS-induced inflammatory responses. After treatment with various GSK-3*β* inhibitors in GAS-infected RAW 264.7 cells, the ratio of nuclear translocation of NF-*κ*B decreased and the levels were even lower than the noninfected group. NF-*κ*B is known to mediate many proinflammatory responses during bacterial infection [46], including GAS [34]. Our results indicate that NF-*κ*B play a central role in regulation of TNF-*α* and iNOS expression. We also found that GSK-3*β*, GAS internalization, and TLR-2 all mediate TNF-*α* and iNOS expression. The detailed mechanism of the interrelationship between GSK-3*β*, GAS internalization, and TLR-2 signaling leading to NF-*κ*B activation remains to be clarified.

In the LPS-induced sepsis model, GSK-3*β* phosphorylation at Ser9 was found to be initially reduced but recovered to baseline level after a short period of time [47–49]. In contrast, our data revealed prolonged dephosphorylation of GSK-3*β* at Ser9 even 24 h after stimulation by GAS. We found that production of TNF-*α* and NO persisted 24 h after infection. TNF-*α* plays a critical role in modulating the cytokine cascade and the fate of macrophages [50]. Deletion of TNF receptor p60 or p80 facilitates LPS-induced apoptosis [51] but desensitizes Fas ligand-induced apoptosis [52]. The dual role of NO in regulation of inflammation is also recognized. Different levels of NO production or the duration to NO exposure are detrimental factors in mediating the degree of inflammation and tissue injury [53]. The pathophysiological role of persistent activation of GSK-3*β* in GAS-stimulated macrophages and the regulation of subsequent inflammatory reactions and cell fate still need further investigation.

Sepsis is a complex interaction between pathogen and host. Sepsis is characterized as the burst production of cytokines, chemokines, and NO. The transcription of these proinflammatory markers occurs mainly through the activation of NF-*κ*B and leads to subsequent tissue hypoperfusion, organ injury, and dysregulation of the coagulation system [54]. In patients with GAS infection, higher circulation level of cytokines, such as TNF-*α* and IL-6, also correlated with the disease severity [10]. Thus, blockade of the activity of NF-*κ*B and the production of proinflammatory mediators might prevent further morbidity and mortality of sepsis. In clinical trials targeting proinflammatory cytokines, such as TNF-*α* or IL-1*β*, in patients with sepsis have not yielded the desirable outcomes [55–57]. In previous studies, the administration of GSK-3*β* inhibitors revealed promising benefits for survival in animal models [16, 20, 23, 36]. In the present study, we also show that GSK-3*β* inhibition provides survival benefit in GAS infection. Live bacterial infection involves the multiplication of bacteria, the production of multiple exotoxins and the various evoked inflammatory pathway after infection. The GAS strain we used is a SPE B-producing strain which could cause the dysregulation of host immune system and facilitate invasion of bacteria [58]. If treatment of GSK-3*β* inhibitors can only suppress the production of proinflammatory cytokines without also blocking SPE B-induced pathogenic effects, persistent replication and dissemination of bacteria would be expected and this will lessen the therapeutic effect of GSK-3*β* inhibitor. However, due to the pleiotropic effect of GSK-3*β* inhibition including anti-inflammation, antiapoptosis, and tissue regeneration [45], GSK-3*β* inhibition may be a superior therapeutic strategy to those utilizing monoclonal antibody antagonists in the management of sepsis in combination with antibiotic treatment. 

## 5. Conclusions

In conclusion, GAS infection in macrophages can mediate the activation of the GSK-3*β*-NF-*κ*B signaling pathway and the subsequent production of TNF-*α* and NO. Inhibition of GSK-3*β* can downregulate NF-*κ*B and its associated inflammatory response. In animal model studies, GSK-3*β* inhibition can reduce TNF-*α* production and lower the mortality when compared to a control group. Since GSK-3*β* inhibition can provide anti-inflammatory effects *in vitro* and *in vivo*, it could offset cytokine storm-related tissue injury during sepsis and might therefore act as an alternative therapeutic strategy beyond antibiotic treatment. 

## Supplementary Material

Supplemental Figure 1. NZ131 induces the activation of GSK-3*β*. Western blot analysis was performed for the dynamic changes of GSK-3*β* activity by determining the level of its specific substrate, phosphorylated GS at Ser641.
Supplemental Figure 2. Heat-inactivated NZ131 mediates slight activation of GSK-3*β* in macrophages. Western blot analysis was used to analyze the effect of heat-inactivated NZ131 (heat-killed, HK; MOI:10) on regulation of GSK-3*β* activity by detecting the expression of phospho-GSK-3*β* at Ser9.
Supplemental Figure 3. GSK-3*β*, GAS internalization, and TLR-2 mediate the production of TNF-*α*. RAW 264.7 cells were treated with NZ131 (MOI:10) in the presence of GSK-3*β* inhibitor LiCl (10 mM), endocytosis inhibitor NH4Cl (10 mM), anti-TLR2 antibodies (2.5 *μ*g/ml), and IgG control (2.5 *μ*g/ml). The cell supernatant was collected 24 h after NZ131 infection and TNF-*α* level was determined by using ELISA kit.
Supplemental Figure 4. GSK-3*β*, GAS internalization, and TLR-2 regulate the expression of iNOS. RAW 264.7 cells were treated with NZ131 (MOI:10) in the presence of GSK-3*β* inhibitor LiCl, endocytosis inhibitor NH4Cl, anti-TLR-2 antibodies, and IgG control. Cell lysate was collected 24 h after NZ131 infection and iNOS expression was determined by Western blot analysis.
