# Supplementary figures and images for: Regulatory Role of GSK-3β on NF-κB, Nitric Oxide, and TNF-α in Group A Streptococcal Infection
Source: Mediators Inflamm. 2013 Mar 5;2013:720689. doi: 10.1155/2013/720689 (PMC3603300; doi:10.1155/2013/720689)

# Supplemental figure 1

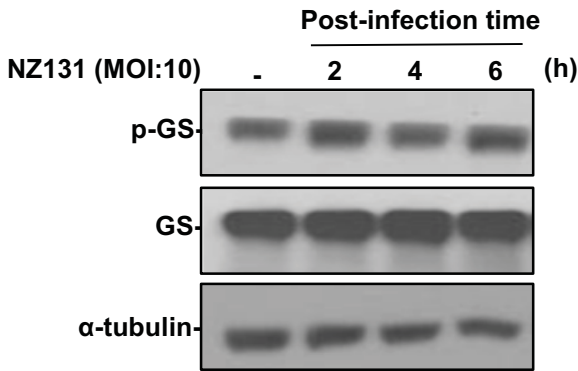

Supplement: Supplementary file 1 — Supplemental Figure 1. NZ131 induces the activation of GSK-3β. Western blot analysis was performed for the dynamic changes of GSK-3β activity by determining the level of its specific substrate, phosphorylated GS at Ser641. Supplemental Figure 2. Heat-inactivated NZ131 mediates slight activation of GSK-3β in macrophages. Western blot analysis was used to analyze the effect of heat-inactivated NZ131 (heat-killed, HK; MOI:10) on regulation of GSK-3β activity by detecting the expression of phospho-GSK-3β at Ser9. Supplemental Figure 3. GSK-3β, GAS internalization, and TLR-2 mediate the production of TNF-α. RAW 264.7 cells were treated with NZ131 (MOI:10) in the presence of GSK-3β inhibitor LiCl (10 mM), endocytosis inhibitor NH4Cl (10 mM), anti-TLR2 antibodies (2.5 μg/ml), and IgG control (2.5 μg/ml). The cell supernatant was collected 24 h after NZ131 infection and TNF-α level was determined by using ELISA kit. Supplemental Figure 4. GSK-3β, GAS internalization, and TLR-2 regulate the expression of iNOS. RAW 264.7 cells were treated with NZ131 (MOI:10) in the presence of GSK-3β inhibitor LiCl, endocytosis inhibitor NH4Cl, anti-TLR-2 antibodies, and IgG control. Cell lysate was collected 24 h after NZ131 infection and iNOS expression was determined by Western blot analysis. [file 720689.f1.pdf]

## Supplemental figure 2

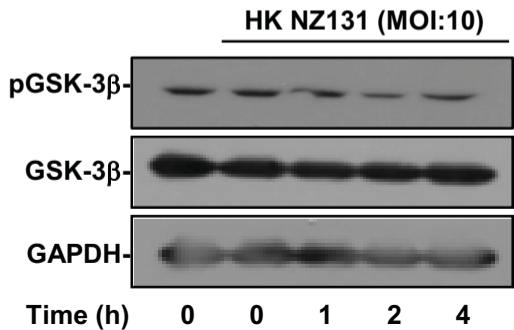

Supplement: Supplementary file 2 [file 720689.f2.pdf]

**Supplemental figure 3**

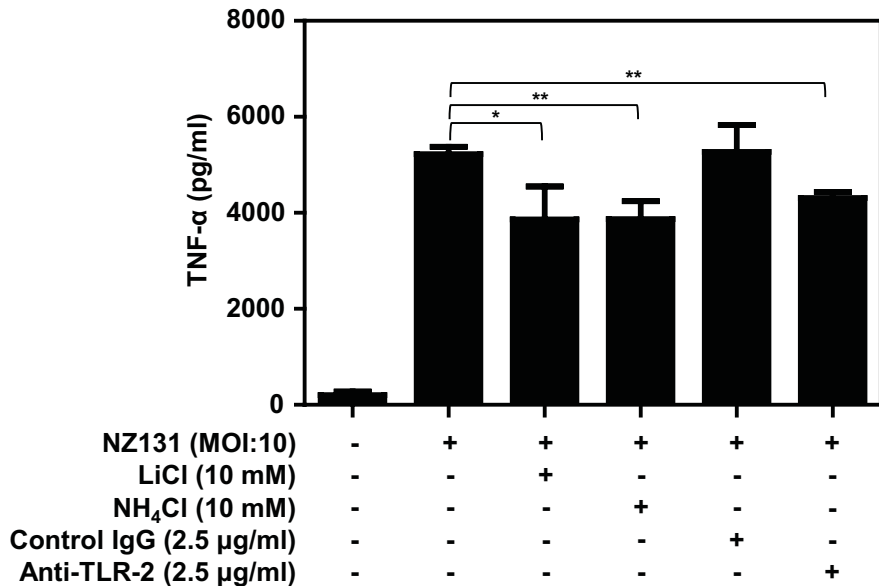

Supplement: Supplementary file 3 [file 720689.f3.pdf]

Supplemental figure 4

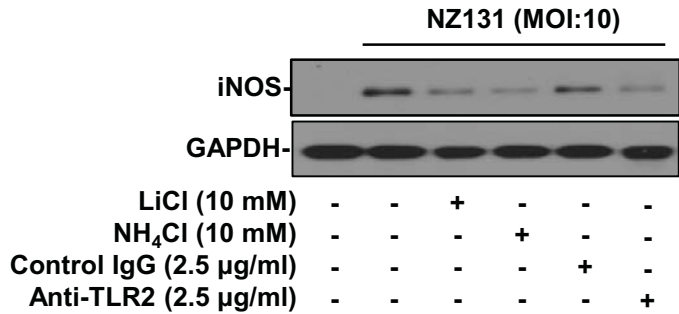

Supplement: Supplementary file 4 [file 720689.f4.pdf]
